# Functionalized monolithic columns: Recent advancements and their applications for high-efficiency separation and enrichment in food and medicine

**DOI:** 10.3389/fchem.2022.951649

**Published:** 2022-08-05

**Authors:** Helong Si, Quan Wang, Yuanyuan Guo, Yuxin Zhao, Hongya Li, Shuna Li, Shuxiang Wang, Baocheng Zhu

**Affiliations:** ^1^ College of Life Science, Hebei Agricultural University, Baoding, Hebei, China; ^2^ Hebei Forage Microbial Technology Innovation Center, Baoding, Hebei, China; ^3^ Hebei Agriculture Waste Resource Utilization Engineering Research Center, Baoding, Hebei, China

**Keywords:** functionalized conolithic column, modification, application, food, medicine

## Abstract

The chromatographic column is the core of a high-performance liquid chromatography (HPLC) system, and must have excellent separation efficiency and selectivity. Therefore, functional modification materials for monolithic columns have been rapidly developed. This study is a systematic review of the recently reported functionalized monolithic columns. In particular, the study reviews the types of functional monomers under different modification conditions, as well as the separation and detection techniques combined with chromatography, and their development prospects. In addition, the applications of functionalized monolithic columns in food analysis, biomedicine, and the analysis of active ingredient of Chinese herbal medicines in recent years are also discussed. Also reviewed are the functionalized monolithic columns for qualitative and quantitative analysis. It provided a reference for further development and application of organic polymer monolithic columns.

## 1 Introduction

As harmful substances continue to affect human health through biochemical reactions, the safety of ingredients in food and medicine has become a focus of increasing attention (Aydoğan et al., 2019; Madikizela et al., 2022). Especially with the improvement of people’s living standards and the advancement of science and technology, growth-promoting hormones, antibiotics, pesticides, food additives, *etc*. are being abused in order to ensure the flavor and output of commodities. Due to incidents, such as the occurrence of mold infestation in Chinese herbal medicines during processing and storage, the presence of above standard levels of mycotoxins in rotting fruits and vegetables and excessive heavy metals in water due to anthropogenic and geogenic pollution, *etc*., the accurate analysis of food and medicine components to detect contaminants that pose significant health risks before entering the market has become a top priority. However, due to the complexity of the matrix and the trace state of residual contaminants, conventional detection methods, such as enzyme-linked immunosorbent assay (ELISA), capillary electrophoresis (CE), and gas chromatography (GC) (Stilo et al., 2021) cannot meet the needs. Therefore, fast, efficient and sensitive liquid chromatography (LC) column detection methods have been widely studied. The use of HPLC for the qualitative and quantitative detection of pharmaceuticals is also a standard analytical technique commonly used in pharmaceutics, and at the same time, it shows its unprecedented advantages in the separation and enrichment of trace mixtures (Zacharis, 2009).

As the core of HPLC, monolithic columns are widely used in fields, such as food (Nunez et al., 2012; Jandera, 2013), medicine (Bunch and Wang, 2011; Memon et al., 2019), environmental (Candel et al., 2009; Liu et al., 2017), proteomics (Meent and de Jong, 2011; Ma et al., 2019), *etc*., due to their advantages of high sensitivity, reusability and eco-friendliness (Miyabe and Guiochon, 2004; Zou et al., 2009; Sharma et al., 2019). In terms of sensitivity, the monolithic column is used for the quantitative and qualitative analysis of active ingredients in foods and medicines, which is more accurate and efficient than other techniques. In terms of safety, the optimized special monomer is more sensitive to trace components in various micron-scale food and drug samples. The organic polymer monolithic column is highly selective, with low mass transfer resistance and high permeability compared to conventional commercial columns. It greatly reduces analysis time while reducing the use of organic solvents. In recent years, the advances of functionalization of monolithic columns have attracted considerable attention. In particular, the improvement of the specific adsorption of monolithic columns, affinity material aptamers and molecular imprinting technique (MIT) have become hot research topics. Monomers have also been developed and used for various purposes, such as nanomaterials to increase the adsorption capacity and mechanical strength, chiral selectors for the resolution of chiral compounds, and ionic liquids (ILs) to improve thermal stability. In order to achieve sensitive and accurate determination of analytes and reduce non-specific adsorption, solid-phase microextraction has been combined with HPLC tandem mass spectrometry (HPLC-MS/MS), LC-MS, capillary electrochromatography (CEC) and other analytical detection methods. The use of early pretreatment greatly reduces the baseline effect of the sample and improves the extraction recovery rate, and in combination with effective detection techniques, the detection effect of two-dimensional (2D)-LC (Jandera et al., 2014; Franco et al., 2016) is achieved.

The application of monolithic columns is also constantly developing and improving. The early stages of their development mainly focused on the detection and enrichment of active components in samples, and later they were widely used in the analysis and quantification of trace harmful residues. This review focuses on the application of organic polymer monolithic columns in food and medicine.

## 2 Functionalization of monolithic columns

### 2.1 Aptamer

Aptamers are synthetic oligonucleotides or peptides with specific secondary and tertiary structures. They were identified *in vitro* through systematic evolution of ligands by exponential enrichment (SELEX) (Zhao et al., 2008). The high specificity of aptamers is achieved by binding to target proteins, and they are used as excellent ligands for biosensors, targeted drug therapy and chromatographic detection (Wang K. et al., 2014; Jiang et al., 2015). Among the various specific adsorption materials, antibodies are easily degraded and expensive, while molecularly imprinted polymers (MIPs) have relatively poor selective adsorption. On the basis of their strong specificity, aptamers have the advantages of ease of synthesis, rapid regeneration, efficient recognition, and target capture (Lyu et al., 2020).

**FIGURE 1:**
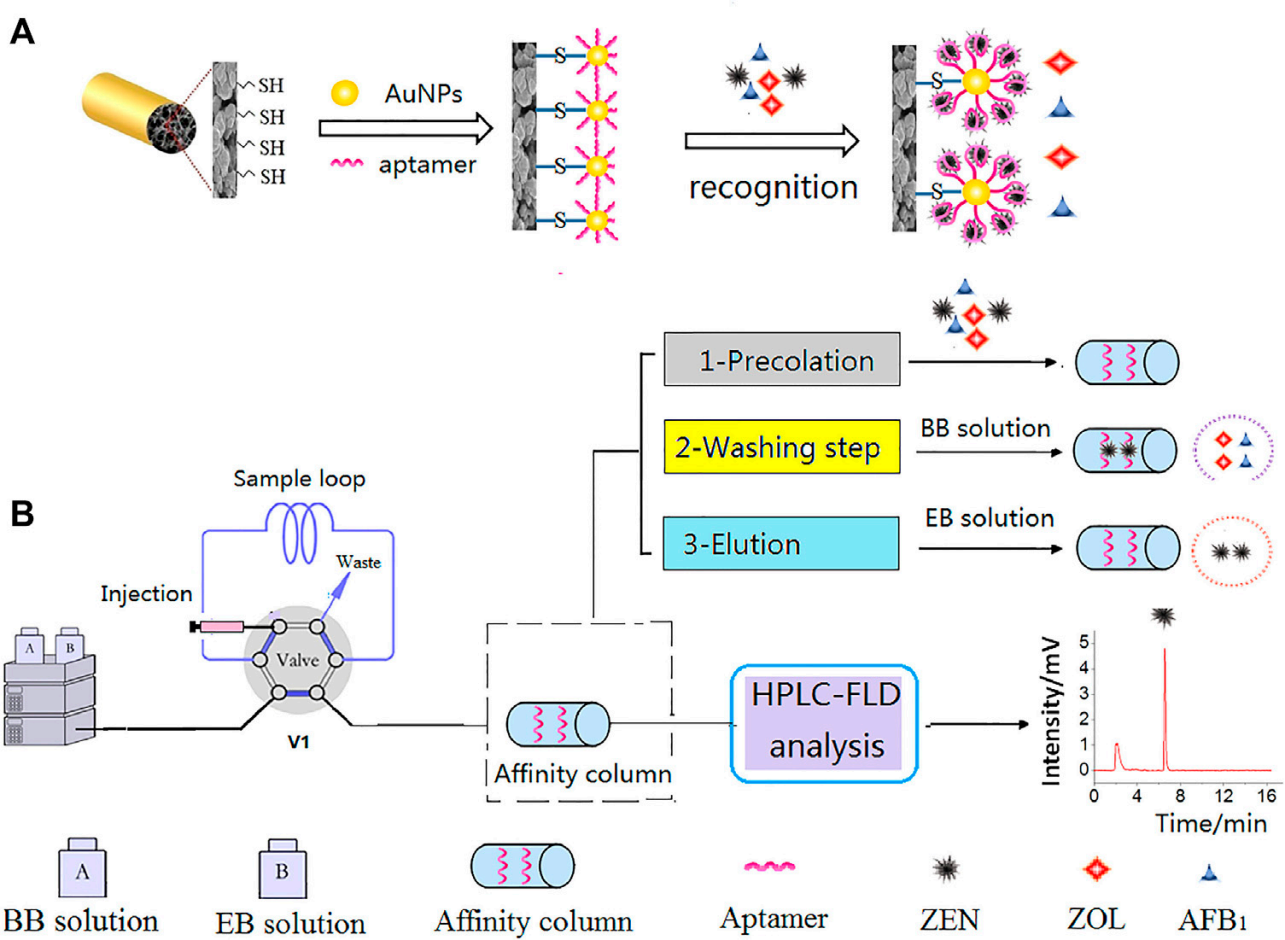
Scheme of preparation of AuNPs@aptamer@ poly (TMOS-co-MPTMS) affinity monolith **(A)** and online selective analysis of ZEN coupled with HPLC **(B)**. Reprinted with permission from Ref. (Xu et al., 2020). Copyright 2020 Elsevier.

In recent years, aptamers have often been used as affinity materials in the preparation of monolithic columns, which are widely used in sample pretreatment and specific detection. In order to avoid the single characteristic of an aptamer, it is often coupled with other functional monomers to obtain a multifunctional affinity monolithic column for the actual preparation. (Xu et al., 2020) exploited the strong conjugation of gold nanoparticles (AuNPs) with the thiol (-SH) functional group to increase the aptamer contact sites. A super-high aptamer coverage density could reach 3,636 pmol μl^−1^. The extraction yield of zearalenone (ZEN) was greatly improved by using hybrid monolithic column with high aptamer density. A preparation and analysis scheme of an aptamer-affinity monolithic column is shown in Figure 1. (Lyu et al., 2020) detected ultratrace amounts of the mycotoxin ochratoxin A in beer, by coupling two selective adsorption materials, namely aptamers and MIPs, which combines the advantages of the two materials, and still maintains high specific adsorption efficiency in the presence of a variety of interference materials.

Aptamers can change the *status quo* that analyzing and detecting trace substances with monolithic columns is difficult. Combining aptamers with other novel functional monomers is also an important research direction.

### 2.2 Nanomaterials

**FIGURE 2:**
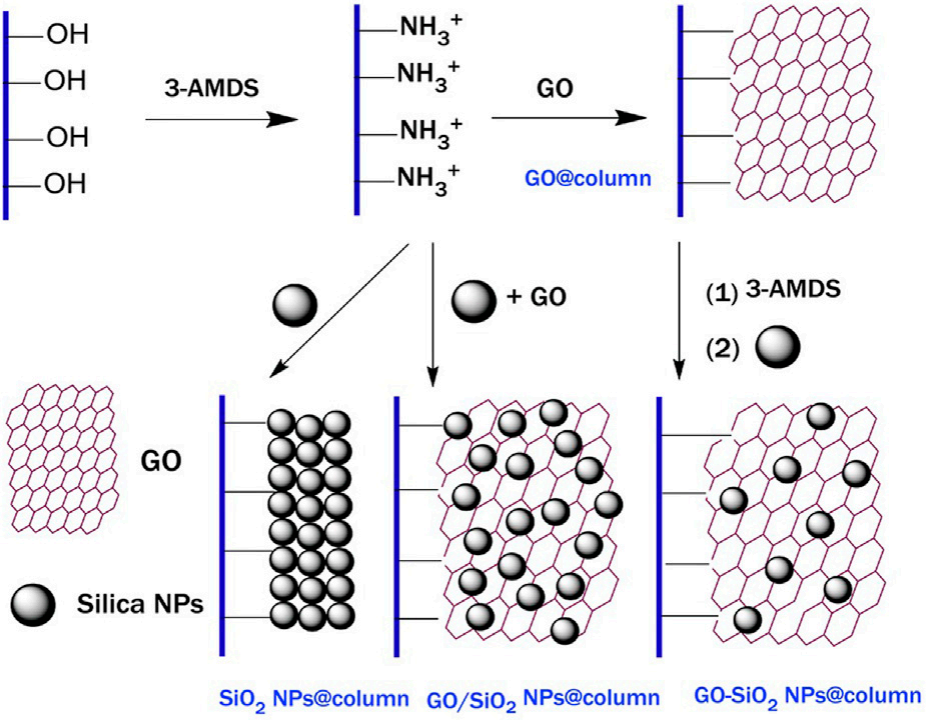
Schematic representation of the fabrication processes of the GO@column, GO/SiO_2_ NPs@column, GO-SiO_2_ NPs@column, and SiO_2_ NPs@column. Reprinted with permission from Ref. (Qu et al., 2016). Copyright 2016 Wiley-VCH.

As a result of their ultra-high specific surface area, mechanical strength and thermal stability, nanomaterials have long been the focus of research in monolithic column chromatography analysis and sample pretreatment (Beeram et al., 2017). Carbon nanotubes are allotropes of carbon and, like graphene, have sp2-hybridized carbon atoms arranged in a honeycomb lattice. They have adsorption characteristics and certain chirality, and can be used as an adsorbent for chromatographic analysis and solid-phase extraction (SPE), or can be added to a monolithic column to achieve enantiomeric separation (Hemasa et al., 2017; Khalil et al., 2020). (Andre and Guillaume, 2012) attached AuNPs to the surface of boron nitride nanotubes (BNNT), thereby significantly improving the retention and separation efficiency of sulfur-based compounds, and demonstrated the performance of BNNT for the first time. For the problems of low solubility and easy agglomeration of graphene oxide (GO), different graphene composite materials have been developed to compensate for these deficits. Additionally (Qu et al., 2016) deposited silica NPs (SiO_2_ NPs) on the surface of GO with a special layered structure. They prepared columns modified with GO (GO@column), GO and SiO_2_ NPs (GO-SiO_2_ NPs@column), and SiO_2_ NPs (SiO_2_ NPs@column), as shown in Figure 2. Their results showed that the column modified with GO/SiO_2_ NPs-C18@column is more efficient and can be applied to the separation of complex samples. Subsequent work has continuously optimized the surface coating of GO for higher column separation efficiency. For instance (Huang L. et al., 2019) used Fe_3_O_4_ grafting to modify GO, which enlarged the gap between GO flakes and provided multiple binding sites for analytes. Compared to traditional imprinting materials, nanostructured imprinting materials are small in size and easy to remove. (Zhai et al., 2015) used the method of coating GO with molecularly imprinted polymers, which not only increased the loading rate of the monolithic column, but also led to the enhancement of the sensitivity for the target.

Nanomaterials form a variety of composite materials through graft modification, which takes advantage of various materials, and increases sample selectivity while increasing monolithic column efficiency.

### 2.3 Chiral selector

There are many chiral compounds on the market today, especially in the pharmaceutical industry, more than half of which are chiral drugs. Commercial chiral columns suffer from high brittleness and manufacturing difficulties. The use of monolithic columns to achieve enantiomeric separation has become a common practice. Due to their good molecular recognition capabilities, polysaccharide derivatives, macrocyclic antibiotics and cinchona alkaloids are often used in the resolution of chiral compounds. For example (Wang et al., 2016) used cinchona alkaloids as chiral selectors to determine the enantiomers of N-derivatized di-and tri-peptides in dietary supplements, and their results show enantiomeric impurities below the limit of detection (LOD). In addition (Zhao et al., 2021) successfully separated ephedrine and pseudoephedrine from ephedra crude extracts using a home-made *ß*-cyclodextrin monolithic column. Based on their verification results, it is reasonable to speculate that there are various interaction forces between the enantiomer and A-*β*-cyclodextrin. (Echevarría et al., 2019) first used the chiral selector cellulose tris (3,5-dimethylphenyl carbamate) in a polymeric monolith column for enantiomeric resolution. The column has enantioselectivity values up to 7.1 and enantioresolutions up to 2.4 in short analysis times. Vancomycin is a macrocyclic antibiotic whose 18 stereocenters and various functional groups are the key to chiral recognition. In another study, a vancomycin derivative was coated on a column to separate the dansyl amino acid enantiomers (Pittler and Schmid, 2010).

Chiral separation can exclude most substances with toxic and side effects, but the application for practical samples is still at an immature stage. The recognition properties and separation mechanisms of chiral monolithic columns still needs to be further investigated.

### 2.4 Ionic liquid

**FIGURE 3:**
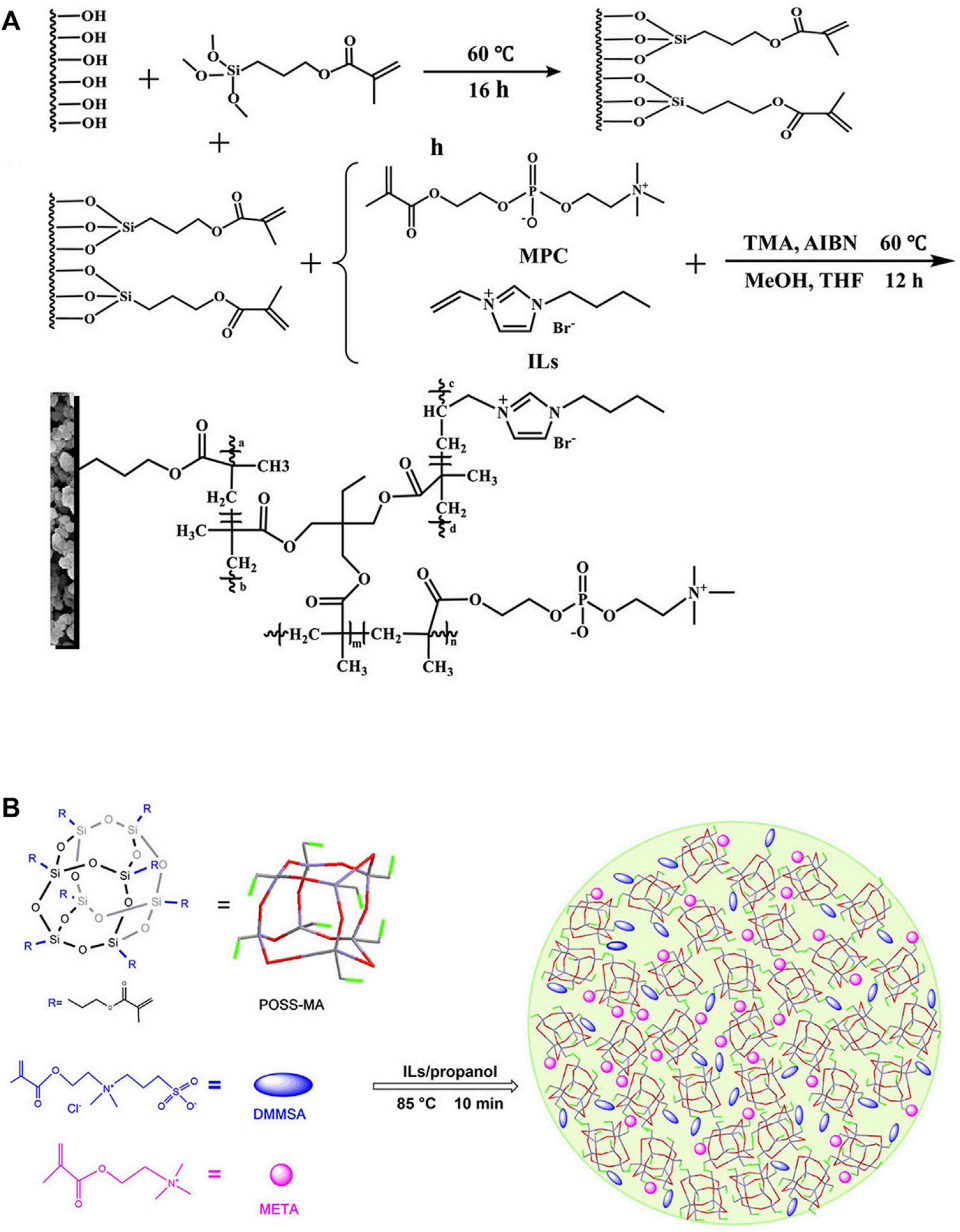
**(A)**: Preparation of poly (MPC-co-ILs-co-TMA) monolithic column. Reprinted with permission from Ref [34]. Copyright 2020 Elsevier. **(B)**: Preparation of poly (POSS-co-META-co-DMMSA) monolithic column. Reprinted with permission from Ref. [37]. Copyright 2021 Elsevier.

As an excellent organic solvent, IL is an excellent solvent for radical polymerization due to its characteristics of high thermal stability, easy adjustment, good high solubility and efficient separation of anions and cations. Compared with traditional ILs, IL monolithic columns can also be obtained by grafting IL molecules onto halide-modified monolithic column materials by nucleophilic substitution. For example, 1-butyl-3-vinylimidazolium bromide (Lei et al., 2020) and pentafluorobenzyl imidazolium bromide IL synthesized by the nucleophilic substitution reaction of 1-vinylimidazole and pentafluorobenzyl bromide (Shan et al., 2015). The IL is often combined with the polymer by methods such as covalent grafting to enhance the stability of the monomer and improve the separation efficiency of the monolithic column (Chen et al., 2019). Phosphorylcholine (ChoP) is an amphiphilic molecule with a high structural similarity to cell membrane components, and is often used in the manufacture of biomimetic medical devices. (Lei et al., 2020) prepared IL monolithic columns by grafting 2-methacryloyloxyethyl phosphorylcholine (MChoP) onto ILs (as shown in Figure 3A), to provide a hydrophilic surface for the stationary phase, while the IL also reduces the defect of poor overall column stability. In some reports, polyhedral oligomeric siloxane (POSS) was introduced into the IL monolithic column (as shown in Figure 3B) to improve the mechanical stability and pH tolerance of the column (Shan et al., 2015; Huang et al., 2021).

ILs have been studied in many aspects due to their unique advantages, but there is still a huge room for development in the exploration of new IL complexes.

### 2.5 Deep eutectic solvent

**FIGURE 4:**
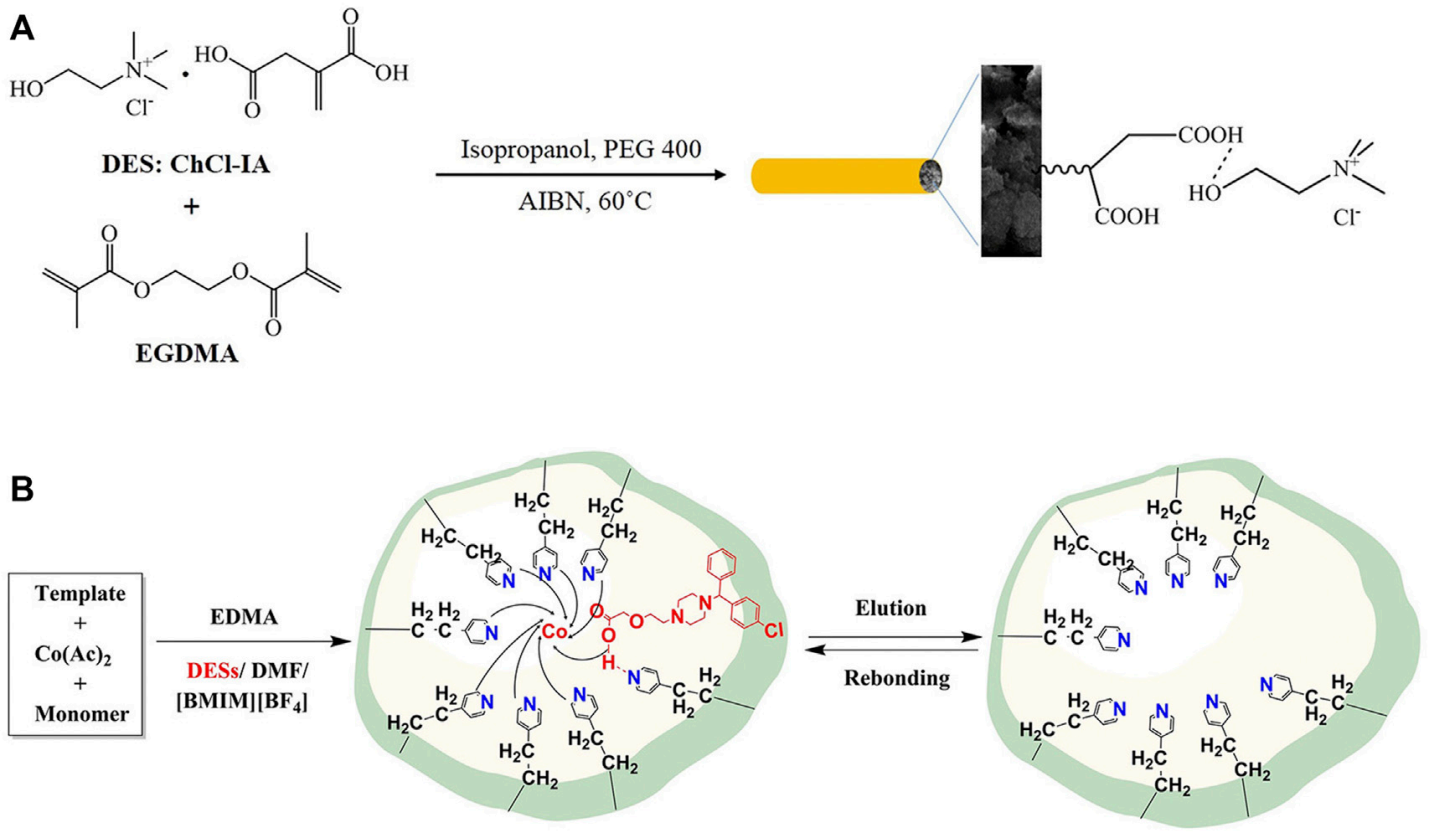
**(A)**: Schematic illustration for fabrication of DES-based monolithic column. Reprinted with permission from Ref. [40]. Copyright 2018 Elsevier **(B)**: The preparation schematic diagram of DES-Co2+-MIP. Reprinted with permission from Ref. [41]. Copyright 2019 Elsevier.

Deep eutectic solvents (DESs) are a new class of green solvents similar to ILs. Compared with ILs, they are low cost, low toxicity and simple to prepare, and have gradually developed into highly effective functional monomers. DESs are a eutectic mixture of a quaternary ammonium salt and a hydrogen bond donor, such as a carboxylic acid, amide or alcohol (Zhang et al., 2016; Wang et al., 2018a). Recently (Wang et al., 2018b) reported the copolymerization of a DES, synthesized from chlorocholine chloride and itaconic acid as monomers, with ethylene glycol dimethacrylate, which formed a novel monolithic column (as shown in Figure 4A). They showed that as a separation column for CEC, the monolithic column can well separate small molecular organisms and alkaloids. Compared with the traditional MIP, the MIP monolithic column prepared with DESs as a porogen has a higher affinity, but also leads to the reduction of imprinted sites. Wei *et al.* (Wei et al., 2019) exploited the high specificity of the metal pivot to compensate for this deficiency, and prepared a metal pivot-bound DES-Co-MIP monolithic column as shown in Figure 4B. The GO modified with 3-(trimethoxysilyl) propylmethacrylate (γ-MPS) was well dispersed in the DESs (choline chloride-alcohol) and room temperature ILs (1-hexyl-3-methylimidazolium tetrafluoroborate), which not only improves the solubility of graphene, but also reduces the interlayer stacking (Li X. X. et al., 2018).

As an easily modified solvent, DESs can be combined with other functional monomers to form copolymerized compounds, bringing out the advantages of each monomer. DESs have great development prospects in chromatographic analysis and detection. The characteristics and shortages of various functional monomers was listed in Table 1.

**TABLE 1:** Characteristics and shortages of each functional monomer.

Functional monomer	Characteristics	Shortages
Aptamer	ease of synthesis, rapid regeneration, efficient recognition, and target capture	fewer contact sites
Nanomaterials	ultra-high specific surface area, mechanical strength and thermal stability	low solubility and easy agglomeration
Chiral Selector	chiral compound resolution	repeatability and service life of chiral monolithic columns low
Ionic liquid	high thermal stability, easy adjustment, good high solubility and efficient separation of anions and cations	exploitation of novel ILs monolithic columns
Deep Eutectic Solvent	low cost, low toxicity, simple to prepare and biocompatible	sticky, uneven distribution of other materials

## 3 Chromatographic detection technology

### 3.1 Molecular imprinting technology

**FIGURE 5:**
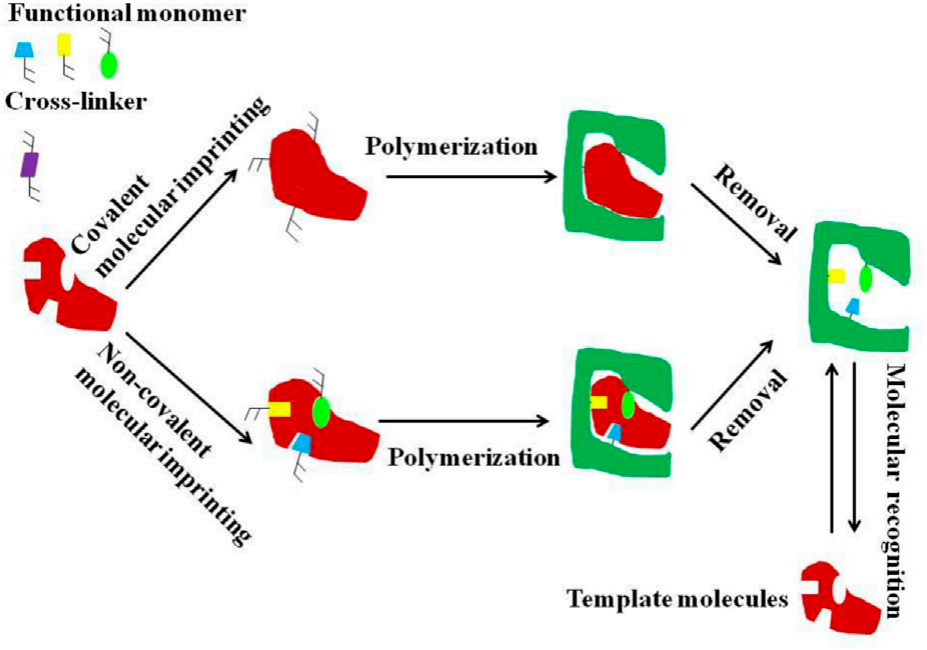
Schematic diagram of molecular imprinting technique. Reprinted with permission from Ref. (Pu et al., 2022). Copyright 2022 Elsevier.

MIT is a technology that can synthesize target molecules by simulating the specific interaction between enzymes and substrates. The principle is that the monomer and the cross-linking agent are copolymerized, and the target molecule is used as a template molecule to combine with the monomer functional group to form a site. When the template molecule is removed, a cavity is formed, and the target molecule is selectively recognized (as shown in Figure 5) (Hermawan et al., 2020; Shen et al., 2021; Pu et al., 2022). A molecularly imprinted polymer (MIP) is synthesized by MIT, and it is used in the separation and detection by integral column chromatography to improve the specific recognition function of the target molecule. The MIP monolithic columns have significant affinity and selectivity for target molecules, but their limited binding sites reduce the column efficiency to a certain extent. In recent years, the high specific surface area of some NPs has attracted considerable attention of researchers. (Ma and Row, 2019) prepared GO-coated molecularly imprinted monolithic columns using ciprofloxacin (CIP) and levofloxacin (LEV) as dual templates, and GO to improve the adsorption capacity and compensate for the low adsorption efficiency. In the practical application of MIP monolithic columns, the problems of low column binding rate and poor repeatability are also due to the incomplete removal of template molecules. This problem can be effectively avoided by preparing the MIPs using the sol-gel method (Moein et al., 2019). (Derazshamshir et al., 2021) designed molecularly imprinted capillary monolithic columns for the separation of the enantiomeric form of the chiral antidepressant S-citalopram (S-CIT) in aqueous solution. Compared with the non-imprinted (NIP S-CIT) monolithic capillary column, the calculated imprinting factor (I.F:1.81) proved the high selectivity of MIP S-CIT monolithic capillary column for S-CIT.

The MIT is a widely used modification technology to improve specific adsorption. The MIT has great potential in sample pretreatment and chromatographic analysis due to its advantages of high selectivity and high specificity.

### 3.2 In-tube solid-phase microextraction

**FIGURE 6:**
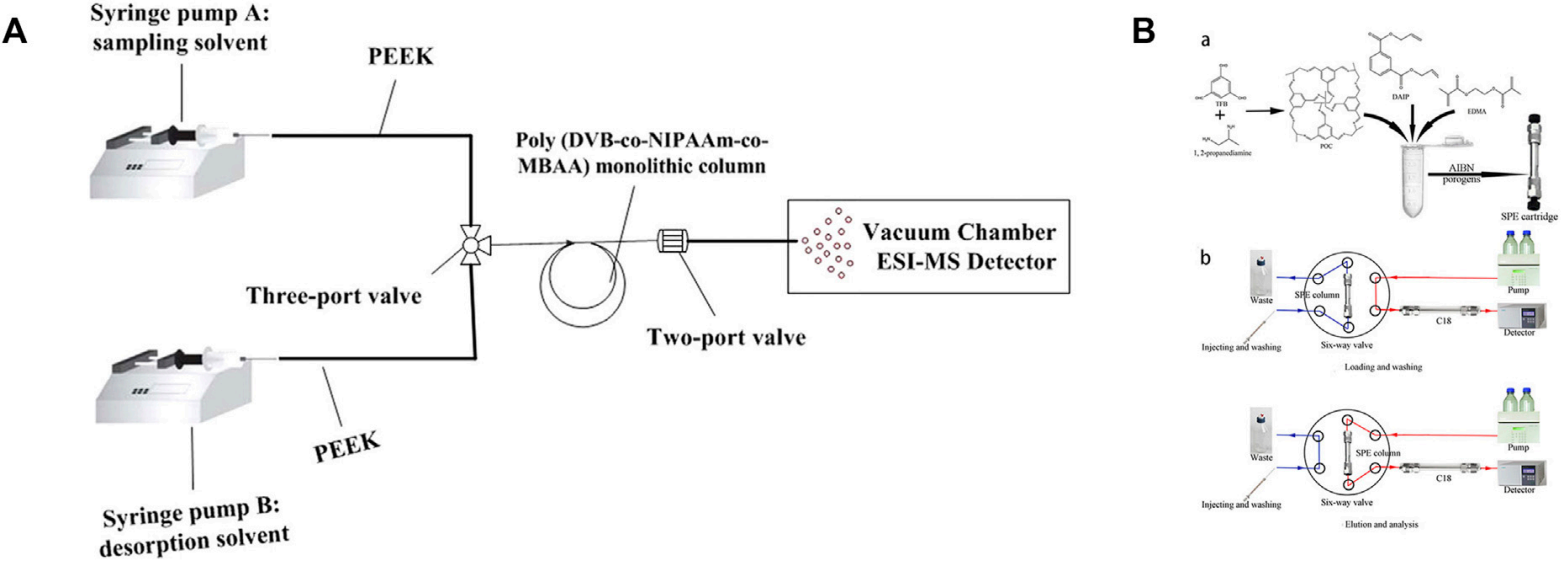
**(A)**: Construction of the online in-tube SPME-MS system. Reprinted with permission from Ref. (Wu et al., 2019). Copyright 2019 Elsevier **(B)**: SPE column synthesis scheme **(A)** and the SPE–HPLC procedures **(B)**. Reprinted with permission from Ref. (Wang L. et al., 2021). Copyright 2021 Wiley-VCH.

The actual sample has complex components, less target molecules, and contains non-volatile endogenous compounds. These compounds are irreversibly adsorbed on the monolithic column, which not only shortens the service life of the monolithic column, but also reduces the analytical sensitivity (Costa Queiroz et al., 2019). Therefore, pretreatment before sample detection becomes an indispensable operation. SPE is the most used traditional sample pretreatment technique. In 1997, Eisert and Pawliszyn proposed the in-tube solid-phase microextraction (SPME) technique in order to couple SPE with HPLC to realize the automation of sample detection (Eisert and Pawliszyn, 1997). In the in-tube SPME technique a capillary column is coated with a capillary surface as an extract. Besides significantly shortening the pretreatment time, this technique reduces the use of organic solvents, and has the advantages of low cost and simple operation. In addition, in-tube SPME can be coupled to various instruments. Especially in combination with HPLC, the sample preparation, separation, and analysis process automation are achieved (Kataoka, 2021). (Wu et al., 2019) coupled in-tube SPME online with HPLC-MS (as shown in Figure 6A), which greatly reduced the influence of baseline effects, and achieved the enrichment of tobacco alkaloids through a simple operation. (Wang K. et al., 2014) installed an optimized SPE column at the six-port valve position as a pretreatment device, connected a C18 column as an analytical column, injected the sample in load mode and eluted the sample matrix with water to complete the sample pretreatment. After pretreatment, the six-port valve was changed to injection mode, and the mobile phase flowed through the SPE column to elute the sample and entered the C18 column for detection and analysis (as shown in Figure 6B). This semi-automated analysis approach greatly reduced time and solvent consumption. (Wu F. et al., 2021) used in-tube SPME-MS for the online detection of benzimidazoles in chicken and pork samples. Their results were more pronounced and had a lower signal-to-noise ratio than MS-only detection.

The overall material is an ideal material for SPE as an extraction adsorbent due to its high permeability. The development of extraction phases with higher selectivity and extraction efficiency and the flexible use of in-tube SPME are future innovations. The achievement of automated, miniaturized, high-throughput instruments and sustainable, green scientific analysis techniques is the future development trend.

### 3.3 Combining multiple technologies

Besides combining various functional monomers to form composite materials, multiple detection technologies are also combined in order to achieve high specificity, high sensitivity and high accuracy detection. SPE is performed before the column is applied to preprocess the sample to remove the complex matrix, but its selectivity is low and the content of the target substance is small. The pretreatment work cannot achieve the desired effect. Therefore, combining SPE with MIT and functionalized monolithic columns to improve the selectivity of pretreatment. (Zhai et al., 2017) used a GO/SiO_2_-MISPE combination chip to efficiently and selectively extract and enrich Rhodamine B in chili powder samples. The combined with nanomaterials enhances the binding ability of template molecules and increases the adsorption area of the monolithic column (Li Y. et al., 2021).

HPLC is often used in combination with MS and fluorescence detection (FLD) to achieve sensitive and accurate results. HPLC-MS/MS is a commonly used method for substance detection with high accuracy and sensitivity. HPLC-FLD has the advantages of high resolution, low cost, and good stability. (Nian et al., 2018) used online SPE combined with online detection by HPLC-MS/MS, which takes 16 min for one analysis and can be repeated 600 times, and indeed achieves fast and efficient automatic detection. This is also the future development direction.

## 4 Applications of polymer monolithic columns in food and medicine

### 4.1 Application in food

#### 4.1.1 Determination of active ingredients

The purpose of detecting active ingredients in food with the monolithic column is twofold, one is to perform high-purity enrichment of the beneficial main active ingredients, and the other is to distinguish adulterants and low-cost food ingredients. For instance, *ß*-sitosterol in plant oil samples has significant anti-inflammatory and antitumor effects, but it is difficult to detect. A new monolithic column was prepared and a new method was developed for online enrichment and determination of *ß*-sitosterol in plant oil samples by online SPE combined with HPLC (Guo et al., 2019; Wang et al., 2019). Phenolic acids have excellent antioxidant activity. (Kuo et al., 2018) used activated carbon polymer monolithic columns to extract phenolic acids in fruit wine and cranberry samples for the first time. Phenolic acid standards showed high extraction recovery rates, ranging from 76.4 to 101% (<3.1% RSDs) and 73.4–100.5% (<4.0% RSDs), respectively. The alkaloids in medicinal and edible plants have great medicinal properties, but plant components seriously interfere with the quantitative analysis due to complex matrices. The poly (tetrahydrofurfuryl methacrylate-co-3,4-ethylenedioxy-N-methylamphetamine-co-triallyl isocyanurate (poly (THFMA-co-EDMA-co-TAIC)) monolith column prepared by the research group of (Li et al., 2019) is a simple, rapid and efficient method for the detection of alkaloids in edible plants. In addition, it has good accuracy and repeatability, and the accuracy illustrated by spiked recovery ranged from 98.89–102.06%. (Pang et al., 2019a) prepared a home-made chromatographic monolithic column for the quantitative determination of six aroma compounds in edible spices, namely, anethole from star anise, trans-cinnamaldehyde from cinnamon, hydroxy-alpha-sanshool from zanthoxylum, as well as 6-gingerol, 8-gingerol, and 10-gingerol from ginger. The detection of real species in different habitats showed that the method has good specificity and durability. Rapid separation and enrichment of fat-soluble vitamins in commercial products have been achieved using HPLC columns, which compared with some traditional methods, is suitable for large-scale analysis with high purity, flexibility and efficiency, and saving time and labor (Kurdi et al., 2017).

#### 4.1.2 Food additives

The abuse and illegal addition of food additives, such as synthetic dyes, nitrates, optical brighteners, *etc*., pose a huge threat to human health. Relevant laws that strictly regulate the types and dosages of food additives have been passed (Wu L. et al., 2021; Cox et al., 2021). For use in the sample analysis step before the product leaves the factory, researchers try to prepare monolithic columns with different functional monomers to optimize the detection efficiency based on the complexity of the sample components. Li *et al.* (Li W. J. et al., 2013) prepared a poly (N-isopropylacrylamide-co-N, N0-methylene bisacrylamide) monolithic column for the determination of food dyes (tartrazine, sunset yellow, allura red, and azorubine) in soft drink samples. The technique introduced γ-alumina NPs, which effectively improved the organic polymer swelling process and low loading. The preparation of a novel IL-modified organic polymer monolithic column for the determination of five acidic food additives in Coca-Cola was recently reported by researchers to have high extraction efficiency, and a recovery rate that reached 85.4–98.3% (Wang T. T. et al., 2014). Additionally (Zhai et al., 2017) developed a solid-phase extraction chip embedded with array monolithic columns of MIP-coated silanized GO (GO/SiO_2_-MISPE), which had higher adsorption capacity, selectivity and affinity than conventional MISPE columns. It was effectively used to eliminate impurities in chili powder and monitor the harmful dye Rhodamine B, with enrichment factor greater than 110 times. Nitrate (NO_3_^−^) and nitrite (NO_2_^−^) have great potential toxicity as food additives and preservatives. Lin *et al.* (Lin et al., 2019) developed a home-made VIM-EDMA monolithic column for the detection of NO_3_^−^and NO_2_^−^ in vegetables, and the sensitivity of this method for the detection of NO_3_^−^ and NO_2_^−^ reached the acceptable daily intake (ADI) levels specified by the European Union.

#### 4.1.3 Mycotoxins

Mycotoxins are toxic secondary metabolites produced by fungi that are usually unavoidable during plant growth and food storage. In order to ensure food safety and human health, current relevant food safety laws and regulations in China limit the maximum residues of mycotoxins in food or raw materials (Stoev, 2015; Avery et al., 2019). (Zhang et al., 2021) used aptamer capillary monolithic columns as specific adsorbents, and ultra-HPLC (UHPLC)-MS/MS technique for the determination of patulin in apple juices. This ultra-sensitive detection method solved the problems of low patulin content and difficult detection in food samples. (Li Q. et al., 2021) prepared a home-made capillary device for instant detection of ochratoxin A consisting of aptamer-functionalized silica photonic crystal microspheres, which could automatically extract target molecules with a sample recovery rate of 86–108%, and its point-of-care analysis could also be expanded for multi-target molecular screening assays. A carbon quantum dot-coated pseudomolecularly imprinted polymer monolithic column, with 5,7-dimethoxycoumarin as the virtual template of aflatoxin B (AFB_1_), was used to selectively enrich and analyze AFB_1_ in peanuts, taking advantage of the high surface area of carbon quantum dots (CDs) to increase molecularly imprinting binding sites, which achieved an enrichment factor of more than 71-fold for this column (Liang et al., 2018).

#### 4.1.4 Antimicrobials and synthetic pesticides

Antibiotics, plant growth regulators, insecticides and disinfectants are used as drugs to promote growth and treatment of animal and plant diseases, and their uncontrolled use has resulted in the proliferation of drug-resistant strains of microorganisms. These antimicrobial drugs not only pollute the environment, but also transfer to the human body through the medium of food, and cause damage to human health. Therefore, it is very important to sensitively and accurately detect the antimicrobial drug residues in food samples (Nasr et al., 2014; Huang L. F. et al., 2019). The common problems in monitoring the analysis of real samples are the matrix complexity of real samples and the difficulty of antibiotic extraction. Liu et al. (2016) effectively reducing the matrix effect by combining the quick, easy, cheap, effective, rugged, and safe (QuEChERS) method with the poly (lauryl methacrylate-co-methacrylic acid-co-ethylene glycol dimethacrylate) poly (LMA-MAA-EDMA) monolithic column to determine chloramphenicol, thiamphenicol and florfenicol in milk and honey. In addition, Lirio *et al.* (Lirio et al., 2016) reduced the complexity of the samples by using aluminum-based metal-organic framework (Al-MOF)-organic polymer monoliths as adsorbents for the SPME of penicillin from river water and milk samples. A novel monolithic capillary column based on a NH_2_-MIL-53(Al) MOF was developed for the determination of eight trace sulfonamides in fish and chicken samples, by combining the advantages of MOF and SPME, and enabled the recovery of sulfonamides with a recovery rate of 85.7–113% (Zhang et al., 2020). Molecular imprinting combines functional monomers, such as acryloyl-*β*-cyclodextrin (A-*β*-CD), which not only improves the selectivity, but also does not significantly reduce the adsorption efficiency. For instance (Liang et al., 2019) and (Zhang et al., 2014) prepared molecularly imprinted monolithic columns for the detection of benzimidazole fungicides in citrus samples and antibacterial agents in enriched meat.

Real samples are complex and variable in the actual detection process. In order to obtain high accuracy, strong specificity, simple and efficient detection results, it is necessary to continuously explore the detection and analysis of organic polymer monolithic columns for daily food produce. The organic polymer monolithic columns have great future potential in food testing, as shown in Table 2 (Li W. J. et al., 2013; Zhang et al., 2013; Wang T. T. et al., 2014; Zhang et al., 2014; Lin et al., 2015; Zhai et al., 2015; Huang et al., 2016; Lirio et al., 2016; Liu et al., 2016; Wang et al., 2016; Chocholouš et al., 2017; Zhai et al., 2017; Zhang et al., 2017; Chen et al., 2018; Liang et al., 2018; Huang L. et al., 2019; Liang et al., 2019; Lin et al., 2019; Wang et al., 2019; Lei et al., 2020; Lyu et al., 2020; Xu et al., 2020; Zhang et al., 2020; Li Q. et al., 2021; Dong et al., 2021; Ganewatta and El Rassi, 2021; Zhang et al., 2021) the application of monolithic columns in food testing has been increasing in recent years.

**TABLE 2:** Application of polymer monolithic columns in food.

Monolithic materials	Initiator	Matrix	Type of column	Analytical method	LOD	Recovery	Ref
Apt-MIP-(POSS-MA)-co-EDMA	DMPA	ochratoxin A in beer	capillary column	SPME + HPLC-FLD	0.07 ng/ml	95.5–105.9%	Lyu et al. (2020)
AuNPS@aptamer-based- poly (TMOS-co-MPTMS)	—	trace ZEN in corn, rice and wheat	capillary column	HPLC-FLD	0.05 ng/ml	91.6–97.8%	Xu et al. (2020)
Fe_3_O_4_/GO- PDTMS/glass array chip	AIBN	TC、CTC and DC in eggs	capillary column	MISPE + HPLC	3.0–5.5 ug/kg	79.7–91.4%	Huang L. et al. (2019)
GO-MISPE	AIBN	phloxine B in coffee bean	capillary column	SPE + HPLC–LIF	0.075 ng/ml	89.5–91.4%	Zhai et al. (2015)
MQD-*co*-HEMA-*co*-EDMA	AIBN	l-carnosine in dietary supplements	capillary column	HPLC	6.25 uM	92–118%	Wang et al. (2016)
MPC-co-ILs-co-TMA	AIBN	GAs from food samples	capillary column	CME-CEC	5.0–10.0 ug/L	76.0–109.7%	Lei et al. (2020)
PDMS-glass chip-GO/SiO2-MISPE	AIBN	rhodamine B in chili powder	capillary column	SPME-HPLC	0.05 ng/ml	83.7–88.4%	Zhai et al. (2017)
NMA-co-DEA-co-EDMA	BPO + DMA	β-sitosterol in six plant oil	stainless steel column	SPE-HPLC	0.006 mg/ml	90.96–103.56%	Wang et al. (2019)
(NIPAAm-*co*-MBAAm)-Al_2_O_3_	AIBN	synthetic food dyes in soft drink	capillary column	PMME-HPLC	9.3–11.5 ng/ml	90.4–109.2%	Li W. J. et al. (2013)
IL- [APMIm]Cl -GMA	DMF	acidic food additives in Coca-Cola beverage	capillary column	In-tube SPME + HPLC	1.2–13.5 ng/ml	85.4–98.3%	Wang T. T. et al. (2014)
VIM-EDMA	AIBN	nitrate and nitrite in vegetables	capillary column	LC-UV	0.06 and 0.05 ug/mL	80.09–107.54%	Lin et al. (2019)
aptamer-AUNPs-SH-GMA-PEGD	AIBN	patulin from apple juice samples	capillary column	SPME + UHPLC-MS/MS	2.17 pmol/L	85.4–106%	Zhang et al. (2021)
aptamer- functionalized SPCMs	—	ochratoxin A in cereal	capillary column	point-of-care analytical device	0.02 ng/ml	86–108%	Li Q. et al. (2021)
CDs-DMIP	AIBN	aflatoxin B_1_ in peanut	capillary column	HPLC-FLD	0.118 ng/ml	79.5–91.2%	Liang et al. (2018)
LMA-MAA-EDMA	AIBN	amphenicol antibiotics in milk and honey	capillary column	QuEChERS + LC-MS/MS	0.02–0.045 ng/g	95.6–100.2%	Liu et al. (2016)
AI-MOF-BMA-EDMA	AIBN	penicillin in milk	capillary column	SPME + UPLC	0.06–0.26 ug/L	89.5–93.5%	Lirio et al. (2016)
AAPBA/MAA-co-EGDMA	AIBN	sulfonamides in fish and chicken	—	—	1.3–4.7 ng/L	85.7–113%	Zhang et al. (2020)
A-β-CD-co-SMWNTs	AIBN	benzimidazole residues in four fruit	capillary column	SPE + HPLC-FLD	0.03–9.68 ng/ml	84.9–98.4%	Liang et al. (2019)
MIMCC-MAA-EGDMA	AIBN	antimicrobials in chicken, pork and egg	capillary column	MIMCC-HPLC	10.0–14.0 ng/L	71.0–108.2%	Zhang et al. (2014)
carbamide-FSNPs-poly (GMM-co-EDMA)	AIBN	food additives, vitamins and biological amines	stainless steel column	HILIC	—	—	Ganewatta and El Rassi, (2021)
ATP-VBIMBr-EDMA	AIBN	PDE-5 inhibitors in functional foods	capillary column	SPME + HPLC-UV	0.5–0.9 ng/ml	95.7–105.7%	Dong et al. (2021)
A-β-CD-silica	AIBN	carbendazim and carbaryl in vegetables	a pipette tip	SPME-HPLC	1.0 and 1.5 ug/kg	92.6–110.1%	Chen et al. (2018)
AB-gel DCP	—	SAs and FWAs in food	capillary column	online enrichment -HPLC	0.05–0.3 and 0.0003–0.001 ug/L	74–113%	Zhang et al. (2017)
cyano monolithic column	—	red colorants in beverages	cyano monolithic column	SIC	0.45 mg/L	—	Chocholouš et al. (2017)
Ag/GO-dual-MISPE-chip	AIBN	bisphenol A and nonyl phenol in fish	capillary column	SPE + HPLC-FLD	2.4 and 4.7 ng/L	83.7–93.2% 85.6–92.4%	Huang et al. (2016)
LMA–MAA–EDMA	AIBN	five aflatoxins and three phenicol antibiotics	capillary column	LC	—	—	Lin et al. (2015)
TEOS-EP	—	*β*-lactam antibiotics in milk and water	stainless steel column	SPE + HPLC	1.5–3 ng/ml	83–105%	Zhang et al. (2013)

3-acrylamidophenylboronic acid (AAPBA); aptamer (Apt); 2, 2edimethoxy2-phenylacetophenone (DMPA); silica photonic crystal microspheres (SPCMs); Polyhedral oligomeric silsesquioxane methacryl substituted (POSS-MA); Glycidyl methacrylate (GMA); poly (ethylene glycol) diacrylate (PEGD); Gold nanoparticles (AuNPs); phosphodiesterase-5 (PDE-5); 1-butyl-3-vinylimidazolium bromide (VBIMBr); glyceryl monomethacrylate (GMM); hydrophilic interaction liquid chromatography (HILIC); fumed silica nanoparticles (FSNPs); 2-Methacryloyloxyethyl phosphorylcholine (MPC); trimethylolpropane trimethacrylate (TMA); ionic liquids (ILs); azobisisobutyronitrile (AIBN); capillary microextraction (CME); capillary electrochromatography (CEC); Glycopeptide antibiotics (GAs); Trimethoxysilylpropanethio (MPTMS); tetramethoxysilane (TMOS); ethylene dimethacrylate (EDMA); high performance liquid chromatography-fluorescence (HPLC-FLD); molecularly imprinted polymers (MIPs); aptamer (Apt); ultra-high performance liquid chromatography-tandem mass spectrometry (UHPLC-MS/MS); ethylene glycol dimethacrylate (EGDMA); methacrylic acid (MAA); solid-phase microextraction (SPME); N-methylolacrylamide (NMA); N,N-diethylacrylamide (DEA); Benzoyl peroxide (BPO); N,N-Dimethylaniline (DMA); solid-phase extraction (SPE); A-β-CD-based MIP, coupled with SMWNTs (β-MMIP); silanized multi-walled carbon nanotubes (SMWNTs); multi-molecularly imprinted olid-phase extraction (MISPE); tetracycline (TC); chlortetracycline (CTC); deoxytetracycline (DC); 1-vinylimidazole (VIM); carbon quantum dots-coated dummy molecularly imprinted (CDs-DMIP); acryloyled *ß*-cyclodextrin (A-*β*-CD); Sequential Injection Chromatography (SIC); sulfonamides (SAs) and fluorescent whitening agents (FWAs); acylhydrazone bond gel (AB-gel); dynamic covalent polymer (DCP); liquid chromatography tandem mass spectrometry (LC-MS/MS); lauryl methacrylate (LMA); quick, easy, cheap, effective, rugged, and safe (QuEChERS); graphene oxide (GO); metal-organic framework (MOF); butyl methacrylate (BMA); lauryl methacrylate (LMA); laser-induced fluorescence (LIF); molecularly imprinted monolithic capillary column (MIMCC); N,N′-methylenebisacrylamide (DMF); 1-Aminopropyl-3-methylimidazolium chloride ([APMIm]Cl); tetraethoxysilane (TEOS); epoxy resin (EP); N,N′-methylene bisacrylamide (MBAAm); N-isopropylacryla-mide (NIPAAm); Polymer monolith microextraction (PMME).

### 4.2 Application in medicine

#### 4.2.1 Detection and analysis of drug levels in humans and animals

**FIGURE 7:**
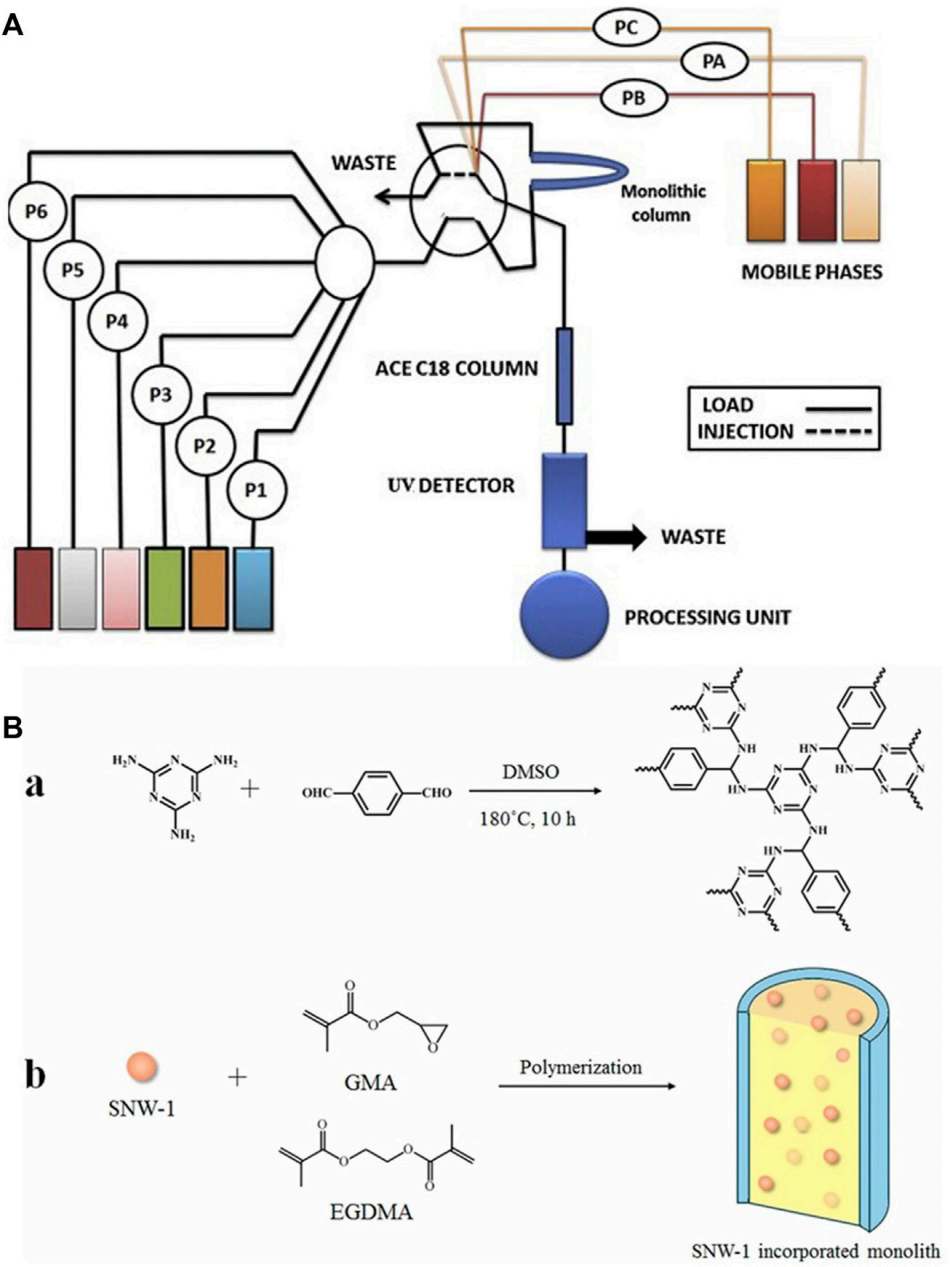
**(A)**: Schematic representation for operation of the on-line assay of the tramadol by monolithic column coupled with HPLC. Reprinted with permission from Ref. (Javanbakht et al., 2012). Copyright 2012 Elsevier **(B)**: Schematic of **(A)** synthesis of SNW-1 and **(B)** preparation of SNW-1-monolithic column. Reprinted with permission from Ref. (Wang R. et al., 2021). Copyright 2021 Elsevier.

With the development of modern scientific and medical technology, people’s health risks from contaminants in food, drinking water, medicines and the enironment have attracted more and more attention. Although current drug treatments can achieve successful outcomes for most human diseases, the pharmacokinetics and pharmacodynamics of each drug are still different among different individuals, and different blood drug concentrations and side effects will also affect people’s health (Tang et al., 2018). Therefore, effective monitoring of drug concentrations in patients is of critical for improving clinical treatment (Novak et al., 2005; Kang et al., 2011). Non-steroidal anti-inflammatory drugs (ketoprofen, fenbufen and ibuprofen) are generally used to treat chronic pain, rheumatism, *etc*. They are over-the-counter drugs without prescription control, but when used for a long time, they may cause gastrointestinal bleeding and cardiovascular disease (Luo et al., 2011; Tang et al., 2018). Lyu *et al.* (Lyu et al., 2015) successfully developed a new type of aluminum terephthalate metal-organic polymer (MIL-53(Al)), and used in polymer monolith microextraction (PMME) capillary monolithic columns for the detection of relatively low concentration of non-steroidal anti-inflammatory drugs in urine, with detection limit and quantification limit reaching from 0.12–0.24 g L^−1^ and 0.40–0.85 g L^−1^, respectively. Javanbakht *et al.* (Javanbakht et al., 2012) used *in situ* MIT to prepare tramadol-imprinted monolithic column in stainless steel tube to directly determine the content of tramadol in human urine and plasma samples. by an online analysis process, which is shown in Figure 7A. Their method achieved detection limits of 0.03 ng ml^−1^ and 0.30 ng ml^−1^, respectively, and recovery rates of 90.5–93.1 and 93.3–96.0%, which effectively prevented the abuse of tramadol. (Wang R. et al., 2021) synthesized a monolithic column incorporating Schiff base network-1 (SNW-1), as shown in Figure 7B, and used it for the determination of three antiepileptic drugs (carbamazepine, oxcarbazepine, lamotrigine) in epilepsy patients, in order to solve the problem that the drug concentration in the plasma cannot be detected in time to prevent the adverse reactions caused by the narrow treatment range of antiepileptic drugs. Its detection limit was 0.2 ng ml^−1^, and the recovery rate was in the range of 88.6–106.1%, indicating that the method has a good application prospect in the extraction and quantitative analysis of antiepileptic drugs. CIP and LEV are second-generation fluoroquinolone antibiotics widely used in the treatment of Gram-negative and Gram-positive bacteria, but their metabolism is slow and prone to allergic reactions and drug resistance (Misurac et al., 2013; Vella et al., 2015; Okan et al., 2017; Tang and Row, 2018). Therefore (Ma and Row, 2019) developed an IL-based, bimolecularly imprinted polymer-coated GO SPE monolithic column for the simultaneous determination of the levels of CIP and LEV in human urine, and achieved recovery rates greater than 93.8% for both antibiotics. Mompo-Rosello *et al.* (Rosello et al., 2020) modified a methacrylate monolithic column with imidazolium-based IL and used it as the stationary phase for SPE to extract five *ß*-blockers from human urine achieving a detection limit of 1.4–40 μg L^−1^, which makes it useful to detect the use of beta-blockers as stimulants. (Lee et al., 2012) used online column conversion ultrafast HPLC-MS-/MS to obtain a good separation of eight barbiturates in human plasma, within 3 min, with a C18 monolithic column, and achieved a quantitative accuracy of 92.0–108%, which is useful in the clinical and toxicological analysis of barbiturates. Chaloemsuwiwattanakan et al. (2016) developed a micro-LC-based synthetic capillary monolithic column for the rapid detection of iodohexanol in human serum, with a detection limit of 0.44 mg L^−1^, and a recovery rate of 102–104%. Effective dose control of iodohexanol and assessment of renal function were reported (Denis et al., 2008; De Baere et al., 2012; Hellqvist et al., 2015).

Also, there are similar problems in animals, and the drug level in animals will directly or indirectly affect the environment and human health. Guo *et al.* (Guo et al., 2017) developed a new molecularly imprinted monolithic column (MIMC) coupled with a 2D-LC (MIMC-2D-LC) method to determine the level of clenbuterol in complex matrices and successfully analyzed clenbuterol in liver and urine samples. Song et al. (2019) used roxithromycin as a virtual template to prepare MIPs of macrolides based on MIP monolithic microextraction (MIPMME) HPLC-MS/MS monolithic column for the analysis of macrolide antibiotic (MAL) residues in pork, chicken and beef samples. Their results showed that MIMCs exhibited better retention capacity for six MALs. Using spiramycin as a virtual template, Zhou et al. (2016) synthesized a molecularly imprinted polymer monolithic column with high selectivity for azithromycin (AZI). They achieved a detection limit and a quantification limit of 0.03 and 0.1 g kg^−1^, respectively, which could effectively monitor residual AZI and its analogues in edible animal tissues to reduce their damage to human liver and heart. In recent years, the application of monolithic columns in medicine has increased, as shown in Table 3 (Xiao et al., 2015; Chen et al., 2016; Wang et al., 2018a; Salih et al., 2018; Peng et al., 2020; Andre and Guillaume, 2021; Derazshamshir et al., 2021; Wang L. et al., 2021; Wu F. et al., 2021; Zhao et al., 2021; Ghanem et al., 2022).

**TABLE 3:** Application of polymer monolithic columns in medicine.

Monolithic materials	Initiator	Matrix	Type of column	Analytical method	LOD	Recovery	Ref
Allyl-β-CD-MMA/TAIC-EDMA	AIBN	crude extract of ephedra, lipopeptide antibiotics	stainless steel column	HPLC	1 ng/ml	100.27–103.77% 97.30–101.33%	Zhao et al. (2021)
poly (DES-EGDMA)	AIBN	NSAIDs in spiked human plasma samples	capillary column	online in-tube SPME-HPLC	0.05–0.5 ng/ml	84.5–105.5%	Wang et al. (2018a)
poly (ethylene glycol dimethacrylate-N-methacryloyl-(L)-phenylalanine methyl ester) (MIP S-CIT)	AIBN	enantioseparation of R,S-citalopram (R,S-CIT) in an aqueous solution	capillary column	CE + HPLC	—	—	Derazshamshir et al. (2021)
DAIP-co-EDMA	AIBN	tussilagone in farfarae flos	stainless-steel column	SPE-HPLC	0.2 μg/ml	100.3–100.6%	Wang L. et al. (2021)
AAPBA-co-DVB-co-MBAA	AIBN	benzimidazoles in animal	capillary column	in-tube SPME-MS	0.55–0.91 ng/g	72.5–92.4%	Wu F. et al. (2021)
poly (GMA-SMX-co-EDMA)	AIBN	eight aromatic ketones and trypsin	capillary column	micro-HPLC	—	—	Xiao et al. (2015)
AM-co-GMA-co-MBA-co-AMPS	AIBN	fifive alkaloids (piperine, nuciferine, kukoline, Berberine, tetrandrine)	capillary column	CEC	0.02–0.1 ug/ml	93.4–108.0%	Chen et al. (2016)
poly (hexyl methacrylate)	AIBN	paracetamol and Chlorzoxazone	capillary column	nano-LC–UV	0.09–0.2 ug/ml	98.32–102.28%	Salih et al. (2018)
(PHEA/TMPTA-EDMA)	AIBN	dioscin in human plasma	stainless steel column	SPE-HPLC-UV	—	96.61–113.73%	Peng et al. (2020)
poly (GMA-EDMA)	—	caffeic acid phenylamide, chlorogenic acid, piceatannol, nor-NOHA acetate	capillary column	HPLC	—	—	Andre and Guillaume, (2021)
polymyxin-B	AIBN	50 racemic pharmaceutical drugs	capillary column	nano-HPLC	—	—	Ghanem et al. (2022)

3-Acrylamidophenylboronic acid (AAPBA); divinylbenzene (DVB); N, N′-Methylenebisacrylamide (MBAA); mass spectrometry (MS); *ß*-cyclodextrin (β-CD); methacrylate (MMA); ethylene dimethacrylate (EDMA); triallyl isocyanurate (TAIC); S-citalopram (S-CIT); R,S-citalopram (R,S-CIT); molecularly imprinted polymer (MIP); paracetamol (PAR); chlorzoxazone (CZN); liquid chromatography (LC); deep eutectic solvent (DES); ethylene glycol dimethacrylate (EGDMA); non-steroidal anti-inflflammatory drugs (NSAIDs); acrylamide (AM); glycidyl methacrylate (GMA); N,N′-methylenebisacrylamide (MBA); 2-acrylamido-2-methyl-1-propane-sulfonic acid (AMPS); capillary electrochromatography (CEC); sulfamethoxazole (SMX); diallyl isophthalate (DAIP); phenyl ether acrylate (PHEA); trimethylolpropane triacrylate (TMPTA).

#### 4.2.2 Analytical applications in chinese herbal medicine

**FIGURE 8:**
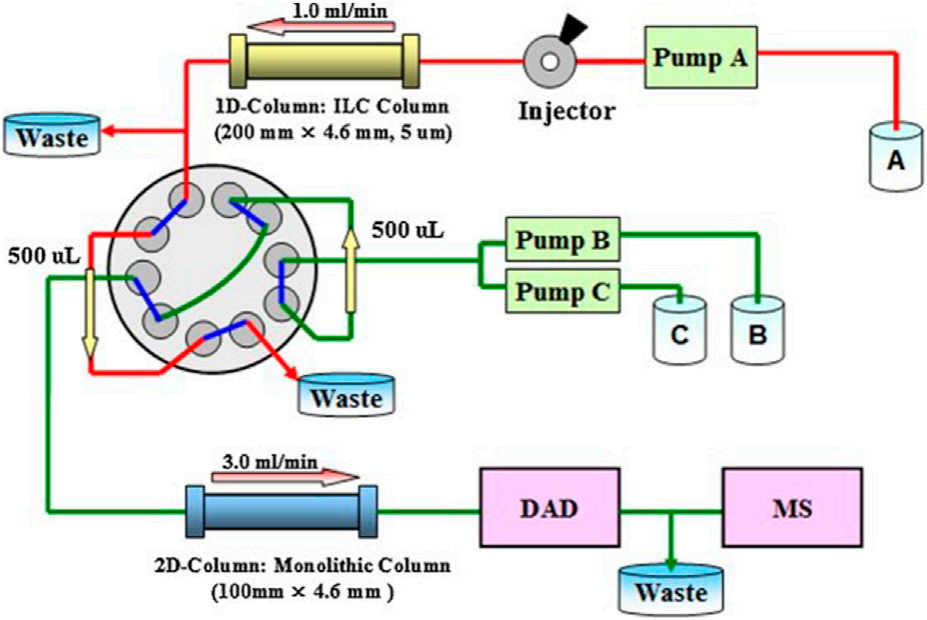
Flowchart of the detection of Schisandra chinensis by immobilized liposome biochromatographic column in two-dimensional chromatography system. Reprinted with permission from Ref. (Wang et al., 2012). Copyright 2012 Elsevier.

Traditional Chinese medicine (TCM) has been widely used in the treatment of various diseases since ancient times. However, TCM is rich in a variety of active components, and the components are similar in structure and very different in content, which makes TCM formulations extremely complex (Zhang et al., 2018; Zheng et al., 2020). It is not easy to accurately separate and analyze each active ingredient. Therefore, before the analysis of TCM samples, more sample pretreatment work is required. The existing methods include liquid-liquid extraction, supercritical fluid extraction, ultrasonic-assisted extraction, microwave-assisted extraction, *etc*. However, most of them require high investment cost, are time-consuming, and their extraction efficiency is low. These inferior properties limit their application. In addition, SPE is also one of the commonly used methods, but its use is also affected by the limited stability of the adsorbent and poor reusability. Consequently, monolithic columns that can be used for trace and structural analysis have been prepared by modifying adsorbents and combining HPLC, MS, and diode array detection (Sun et al., 2021). The Chinese medicine *Schisandra* has antioxidant, detoxification, anticancer and anti-fatigue properties (Wang et al., 2012; Li Z. et al., 2018) developed a biochromatographic column using immobilized liposomes as a membrane model, and separated more than 40 components of *Schisandra chinensis* using a 2D chromatography system (shown in Figure 8), and preliminarily established a three-dimensional chromatographic fingerprint of *Schisandra chinensis*. Sun et al. (2021) prepared a porous organic polymer/vinylic-functionalized covalent organic framework (POP/V-COF) monolithic column with large specific surface area and good stability through a one-step reaction, and used it with a C18 chromatographic column to determine the four active components in a Danshen drink decoction online. They achieved a LOD and a limit of quantification (LOQ) for the four active ingredients of 15–30 and 50–100 ng ml^−1^, demonstrating that these columns are useful for the quality evaluation of TCM formulations. Five kinds of coumarins (latemycin, oxyomycin, xanthoxin, 5-hydroxy-8-methoxypsoralen) in *Angelica* extract have been reported to show anti-inflammatory, analgesic, efficacy in the treatment of abnormal pigmentation disorders and other biological activities (Matsuo et al., 2020; Guo et al., 2021; Yang et al., 2021; Yuan et al., 2022). (Chen Z. et al., 2012) synthesized a methacrylate monolithic column and used sodium deoxycholate and a silver daily mobile phase to achieve the baseline separation of five structurally similar coumarins within 6 min, with recovery rates ranging from 87.5–95.0%, a LOD below 0.15 g ml^−1^, and a LOQ below 0.30 g ml^−1^. (Pang et al., 2019b) prepared MOF-polymer monolithic columns using modified MOF materials and N-methacrylamide as monomers. They were able to extract and purify triterpenoid ursolic acid from Chinese herbal medicine samples with complex matrices, with a LOD and a LOQ of 0.17 μg ml^−1^ and 0.57 μg ml^−1^, respectively, indicating that they can be applied to the determination and enrichment of ursolic acid in routine laboratory TCM samples. (Wang and Shen, 2019) synthesized a new type of porous monolithic column using the original free radical, exceeding the theoretical plate number of 31,000 plates m^−1^, and separated the six main drugs in the TCM *Panax notoginseng* within 10 min (Chen X. et al., 2012) developed a novel 2D HepG_2_/ceramic matrix composite (CMC)/monolithic column/time of flight (TOF)-MS (2D HepG_2_/CMC/monolithic column/TOF-MS) system, by applying monolithic columns to the second dimension (HPLC or GC-MS) for offline or online analysis of one-dimensional retained fractions. Screening of antitumor components of berberine, tetrahydropalmitine, baicalin oxymatrine and matrine, revealed a time-saving, efficiency-enhancing new method for lead discovery.

#### 4.2.3 Analytical application in the separation and detection of enantiomeric drugs

**FIGURE 9:**
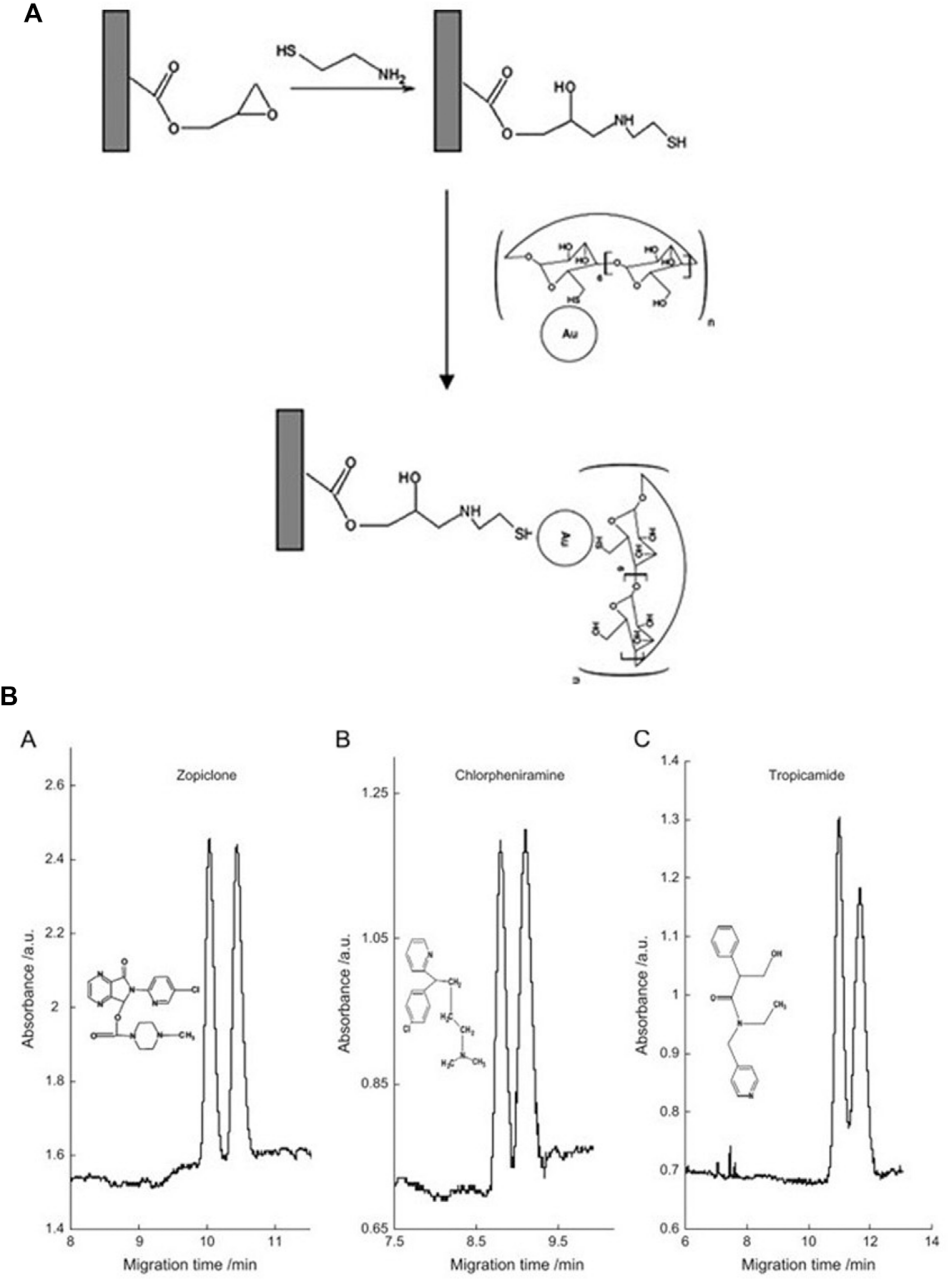
**(A)**: Reaction scheme for the fabrication of the CD-GNP-modified monolithic column **(B)**: Electropherograms of separations of three drug enantiomer pairs using the CD-GNP-modified monolithic columns. A 25 mM phosphate buffer (pH 3.0) was used as the running buffer. The separation electric field strength was 312.5 V/cm. Detection was carried out on-column at 214 nm. Samples were injected electrokinetically at 5 kV for 3 s. Reprinted with permission from Ref. (Li M. et al., 2013). Copyright 2013 Elsevier.

**FIGURE 10:**
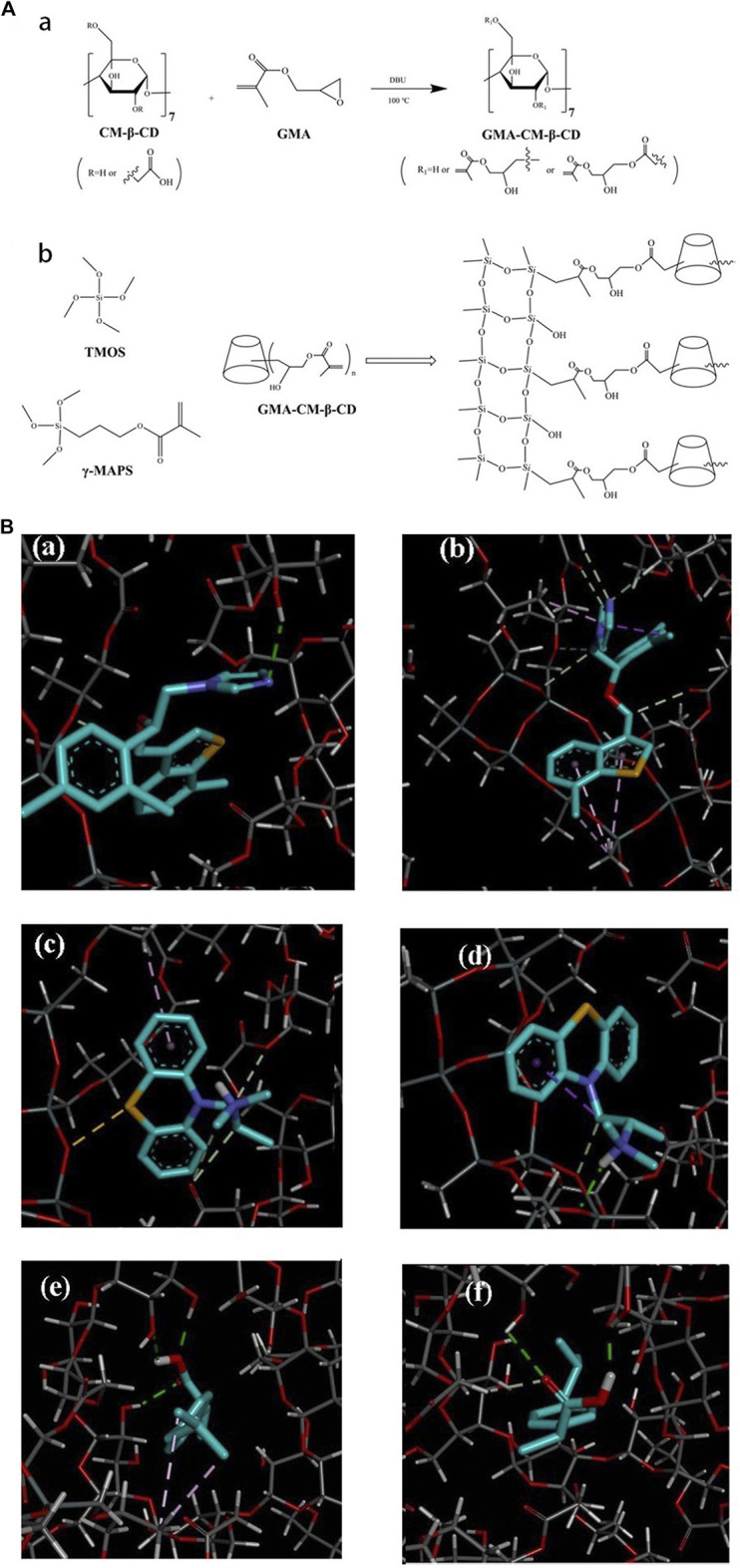
(Continued).

A pair of enantiomeric drugs usually exhibit similar physicochemical properties (*e.g*., color, odor, solubility), but have different physiological activities, and some may have severe toxic side effects, like thalidomide (Svec, 2005; Sun et al., 2019). Therefore, good chiral separation of enantiomeric drugs is essential. Rocco *et al.* (Barnes et al., 2013) performed baseline separation of the enantiomers of nomifensine and naproxen by synthesizing a hydroxypropyl-β-cyclodextrin (HP-β-CD) capillary monolith. (Li M. et al., 2013) used *ß*-cyclodextrin-modified AuNPs (CD-AuNPs) monomers as the stationary phase (the modification scheme of CD-GNP is shown in Figure 9A) for CEC to perform baseline separation of three pairs of drug enantiomers (chlorpheniramine, zopiclone, and tropicamide), with a resolution of 1.85 and their experimental results are shown in Figure 9B. Bao et al. (2021) prepared an anionic carboxymethyl-*β*-cyclodextrin (CM-*β*-CD) silica hybrid monolithic column (preparation process is shown in Figure 10A) by a “one-pot” method for the chiral separation of 10 racemic compounds. Their recovery rates reached 96.79–97.88%, and the column was successfully applied to the detection of enantiomeric impurities of S-ofloxacin, and the molecular recognition mechanism is shown in Figure 10B. Dixit and Park, (2015) synthesized derivatives of zirconium tetraoxide and erythromycin of 3-triethoxysilylpropyl carbamoylated derivative of erythromycin (TEOSPC-ERY) by an *in situ* sol-gel method. They used the organic-inorganic mixed monolithic column to separate six basic chiral drugs, and the highest resolution obtained was Rs = 3.33. Thus, the above studies showed that monolithic columns have good application prospects as a stationary phase in LC to separate chiral drugs, and may be a very useful quality control tool for evaluating enantiomeric drugs.

## 5 Conclusion and prospects

In the continuous improvement and optimization, monolithic columns still have a huge development potential, which is worthy of further exploration. In this review, the different modified materials of monolithic columns and their advantages were reviewed, revealing that they are still not competitive compared to traditional commercial silica monolithic columns and cannot be put into use in large quantities (Guiochon, 2007). First, the stability and repeatability of organic polymer monolithic columns cannot achieve the effect of batch preparation, which is also for a problem limiting its commercial use. Second, the practical application of monolithic columns is complex and diverse, and whether it can meet the standards of practical needs is still an issue to be solved. The monolithic column is used in two dimensions, the front part is pretreated by SPME, and the latter part achieves the effect of sample detection. This kind of separation, analysis and detection that tends to be automated should be the focus of future development. The monolithic column is directionally functionalized as required to prepare a functionalized monolithic column with directional separation characteristics, high separation efficiency, batch preparation, and green friendly, which still requires continuous research and exploration by scientific researchers.

## Abbreviations

CE, capillary electrophoresis; CEC, capillary electrochromatography; DESs, Deep eutectic solvents; ELISA, enzyme-linked immunosorbent assay; GC, gas chromatography; HPLC, high-performance liquid chromatography; HPLC-MS/MS, HPLC tandem mass spectrometry; HPLC-FLD, HPLC-fluorescence detection; LC, liquid chromatography; LOQ, limit of quantification; LOD, limit of detection; MIT, molecular imprinting technique; MIPs, molecularly imprinted polymers; ILs, ionic liquids; SPE, solid-phase extraction; SPME, solid-phase microextraction; TCM, Traditional Chinese medicine.
